# Supplementary material for: No association of COMT with insight problem solving in Chinese college students
Source: PeerJ. 2019 Apr 15;7:e6755. doi: 10.7717/peerj.6755 (PMC6472467; doi:10.7717/peerj.6755)
Supplement: Supplemental Information 1 — The ten classic insight problems (five classic verbal insight problems and five classic figural insight problems) used in this study. [file peerj-07-6755-s002.doc]

**Appendix A**

**Insight Problems Used in this study**

1. A prisoner was attempting to escape from a tower. He found in his cell a rope, which was half long enough to permit him to reach the ground safely. He divided the rope in half and tied the two parts together and escaped. How could he have done this?

2. How can you arrange 6 identical pencils in such as way as to form 4 identical triangles whose sides area are all equal, without modifying the pencils in any way?


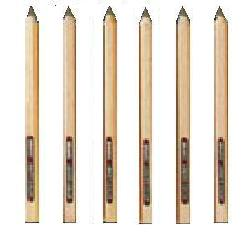


3. Xiao Ming and Xiao Qiang were born on the same day of the same month of the same year to the same mother and the same father - yet they are not twins. How is that possible?

4. There are two strings hanging from the ceiling in the room below. The woman cannot reach both. How can she tie the two strings together?


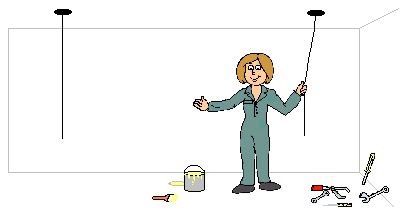


5. The legendary runner Flash Fleetfoot was so fast that his friends said he could turn off the light switch and jump into bed before the room got dark. On one occasion Flash proved he could do it. How?

6. The triangle shown below points to the top of the page. Show how you can move three circles to get the triangle to point to the bottom of the page.

7. A magician claimed to be able to throw a ping pong ball so that is would go a short distance, come to a dead stop, and then reverse itself. He also added that he would not bounce the ball against any object or tie anything to it. How could he perform this feat?

8. Given 4 matches arranged to represent a glass and a dot representing a coin the glass move 2 matches so coin is outside the glass

**￥1**

9. Xiao Hong stumbles across an abandoned cabin one cold, dark and snowy night. Inside the cabin is a kerosene lantern, a candle, and wood in a fireplace. She only has one match. What should she light first?

10. Transform an arrangement of 8 coins into one where each coin touches exactly 3 others.

**￥1**

**￥1**

**￥1**

**￥1**

**￥1**

**￥1**

**￥1**

**￥1**
